# Supplementary material for: Variable patterns of mutation density among NaV1.1, NaV1.2 and NaV1.6 point to channel-specific functional differences associated with childhood epilepsy
Source: PLoS One. 2020 Aug 26;15(8):e0238121. doi: 10.1371/journal.pone.0238121 (PMC7449494; doi:10.1371/journal.pone.0238121)
Supplement: S1 Fig — Brown lines indicate protein boundaries. (DOCX) [file pone.0238121.s001.docx]

**S1 Fig.** Cumulative distribution plot comparing public and patient Na_V_1.2 variants. Brown lines indicate protein boundaries.

**
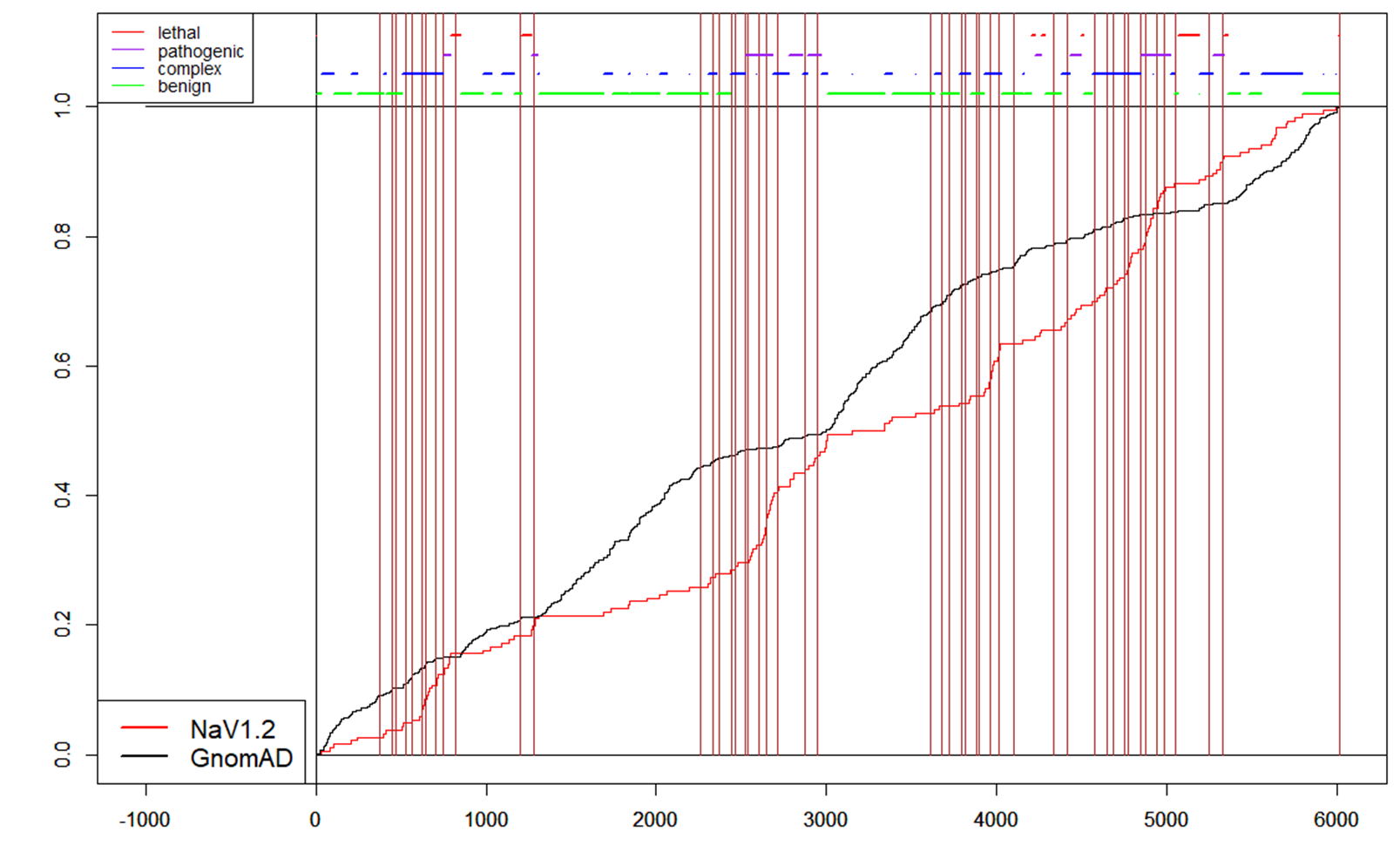
**
