# Supplementary material for: Variable patterns of mutation density among NaV1.1, NaV1.2 and NaV1.6 point to channel-specific functional differences associated with childhood epilepsy
Source: PLoS One. 2020 Aug 26;15(8):e0238121. doi: 10.1371/journal.pone.0238121 (PMC7449494; doi:10.1371/journal.pone.0238121)

**S4 Fig**. Intolerance graph. The –log p-value of the computed z-scores for the nine VGSCs was calculated and were grouped based on their values. The sodium channel number represents the SCN gene to which each value corresponds.


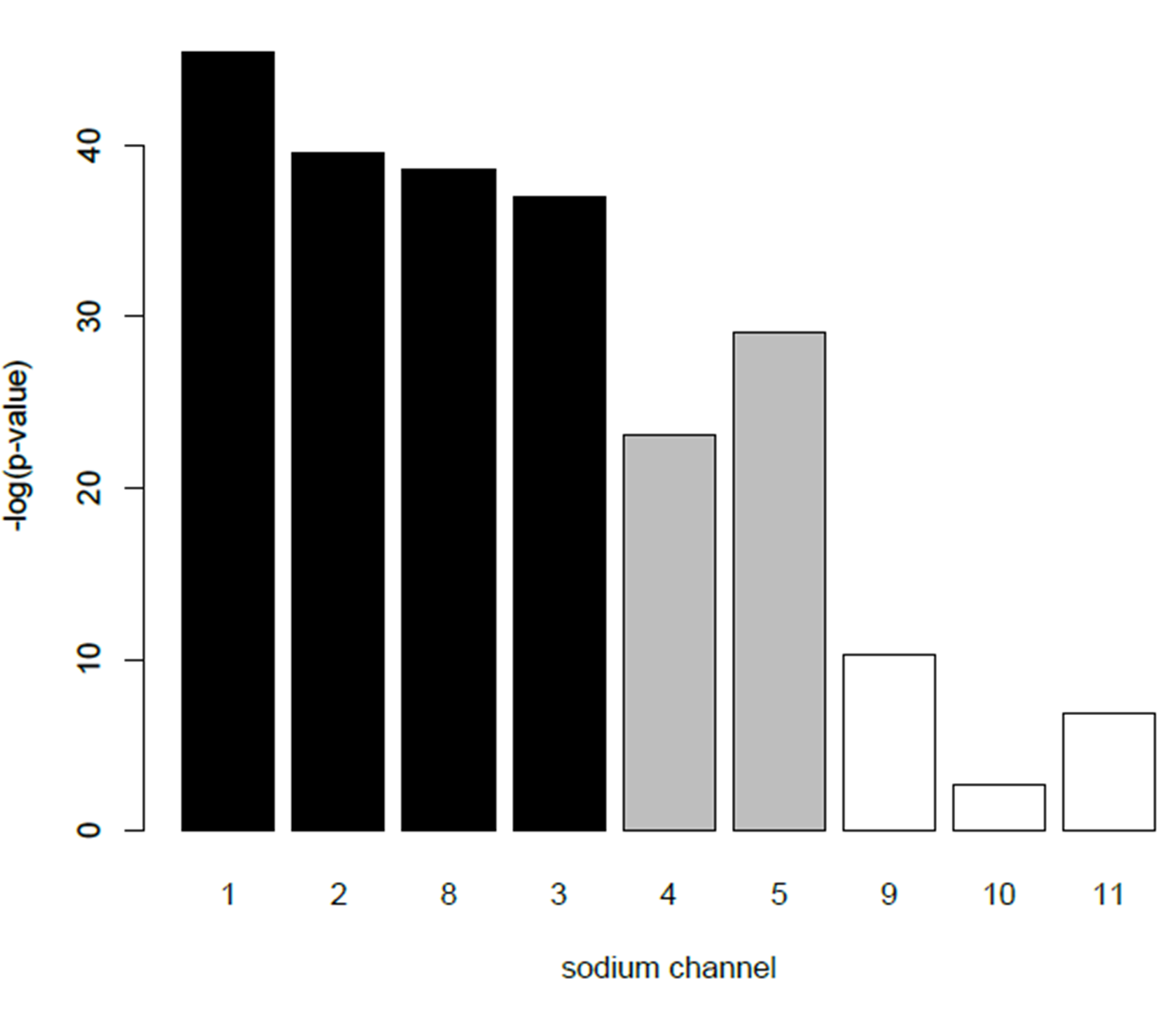

Supplement: S4 Fig — The–log p-value of the computed z-scores for the nine VGSCs was calculated and were grouped based on their values. The sodium channel number represents the SCN gene to which each value corresponds. (DOCX) [file pone.0238121.s004.docx]
