# Supplementary material for: Variable patterns of mutation density among NaV1.1, NaV1.2 and NaV1.6 point to channel-specific functional differences associated with childhood epilepsy
Source: PLoS One. 2020 Aug 26;15(8):e0238121. doi: 10.1371/journal.pone.0238121 (PMC7449494; doi:10.1371/journal.pone.0238121)
Supplement: S1 Table — (DOCX) [file pone.0238121.s005.docx]

**S1 Table**. List of all patient and public (GnomAD) mutation numbers for each Na_V_ channel.

| Na_V_ Channel | Patient (n) | GnomAD (n) |
| --- | --- | --- |
| 1.1 | 557 | 619 |
| 1.2 | 198 | 489 |
| 1.6 | 124 | 390 |
| 1.3 | - | 679 |
