# Supplementary material for: Variable patterns of mutation density among NaV1.1, NaV1.2 and NaV1.6 point to channel-specific functional differences associated with childhood epilepsy
Source: PLoS One. 2020 Aug 26;15(8):e0238121. doi: 10.1371/journal.pone.0238121 (PMC7449494; doi:10.1371/journal.pone.0238121)
Supplement: S2 Table — (DOCX) [file pone.0238121.s006.docx]

**S2 Table**. List of patient variants used for these analyses.

| **Na_V_ Channel** | **Nucleotide change** | **Protein change** | **Na_V_ Channel** | **Nucleotide change** | **Protein change** | **Na_V_ Channel** | **Nucleotide change** | **Protein change** |
| --- | --- | --- | --- | --- | --- | --- | --- | --- |
| NaV1.1 s | c.70G>A | p.Ala24Thr | NaV1.1 s | c.4243T>A | p.Phe1415Ile | NaV1.2 | c.787G>A | p.Ala263Thr |
| NaV1.1 s | c.80G>C | p.Arg27Thr | NaV1.1 s | c.4246G>C | p.Asp1416His | NaV1.2 | c.788C>T | p.Ala263Val |
| NaV1.1 s | c.173G>T | p.Gly58Val | NaV1.1 s | c.4247A>G | p.Asp1416Gly | NaV1.2 | c.982T>G | p.Phe328Val |
| NaV1.1 s | c.181C>T | p.Leu61Phe | NaV1.1 s | c.4250A>G | p.Asn1417Ser | NaV1.2 | c.1028A>G | p.Asp343Gly |
| NaV1.1 s | c.187T>C | p.Phe63Leu | NaV1.1 s | c.4253T>A | p.Val1418Glu | NaV1.2 | c.1094C>T | p.Thr365Met |
| NaV1.1 s | c.203T>C | p.Ile68Thr | NaV1.1 s | c.4253T>G | p.Val1418Gly | NaV1.2 | c.1136G>A | p.Arg379His |
| NaV1.1 s | c.226C>A | p.Pro76Thr | NaV1.1 s | c.4265A>G | p.Tyr1422Cys | NaV1.2 | c.1165C>T | p.Leu389Phe |
| NaV1.1 s | c.234G>T | p.Glu78Asp | NaV1.1 s | c.4267C>T | p.Leu1423Phe | NaV1.2 | c.1264G>C | p.Ala422Pro |
| NaV1.1 s | c.235G>A | p.Asp79Asn | NaV1.1 s | c.4277T>G | p.Leu1426Arg | NaV1.2 | c.1267G>C | p.Val423Leu |
| NaV1.1 s | c.235G>C | p.Asp79His | NaV1.1 s | c.4280A>C | p.Gln1427Pro | NaV1.2 | c.1270G>C | p.Val424Leu |
| NaV1.1 s | c.243C>A | p.Asp81Glu | NaV1.1 s | c.4291T>A | p.Phe1431Ile | NaV1.2 | c.1270G>A | p.Val424Met |
| NaV1.1 s | c.251A>G | p.Tyr84Cys | NaV1.1 s | c.4294A>G | p.Lys1432Glu | NaV1.2 | c.1288G>C | p.Glu430Gln |
| NaV1.1 s | c.272T>C | p.Ile91Thr | NaV1.1 s | c.4295A>G | p.Lys1432Arg | NaV1.2 | c.1289A>C | p.Glu430Ala |
| NaV1.1 s | c.278T>C | p.Leu93Ser | NaV1.1 s | c.4297G>A | p.Gly1433Arg | NaV1.2 | c.1289A>G | p.Glu430Gly |
| NaV1.1 s | c.282T>A | p.Asn94Lys | NaV1.1 s | c.4298G>A | p.Gly1433Glu | NaV1.2 | c.1307T>C | p.Leu436Ser |
| NaV1.1 s | c.292G>C | p.Ala98Pro | NaV1.1 s | c.4298G>T | p.Gly1433Val | NaV1.2 | c.1691G>T | p.Gly564Val |
| NaV1.1 s | c.301C>T | p.Arg101Trp | NaV1.1 s | c.4300T>C | p.Trp1434Arg | NaV1.2 | c.1737C>G | p.Ser579Arg |
| NaV1.1 s | c.302G>A | p.Arg101Gln | NaV1.1 s | c.4311A>G | p.Ile1437Met | NaV1.2 | c.1835T>C | p.Phe612Ser |
| NaV1.1 s | c.307A>G | p.Ser103Gly | NaV1.1 s | c.4313T>C | p.Met1438Thr | NaV1.2 | c.1840C>T | p.Pro614Ser |
| NaV1.1 s | c.311C>T | p.Ala104Val | NaV1.1 s | c.4319C>A | p.Ala1440Glu | NaV1.2 | c.1945G>A | p.Asp649Asn |
| NaV1.1 s | c.314C>T | p.Thr105Ile | NaV1.1 s | c.4321G>C | p.Ala1441Pro | NaV1.2 | c.2021C>A | p.Thr674Lys |
| NaV1.1 s | c.320C>A | p.Ala107Asp | NaV1.1 s | c.4321G>A | p.Ala1441Thr | NaV1.2 | c.2063A>G | p.Tyr688Cys |
| NaV1.1 s | c.323T>G | p.Leu108Arg | NaV1.1 s | c.4348C>A | p.Gln1450Lys | NaV1.2 | c.2197G>A | p.Ala733Thr |
| NaV1.1 s | c.335C>T | p.Thr112Ile | NaV1.1 s | c.4349A>G | p.Gln1450Arg | NaV1.2 | c.2306T>C | p.Ile769Thr |
| NaV1.1 s | c.337C>A | p.Pro113Thr | NaV1.1 s | c.4349A>C | p.Gln1450Pro | NaV1.2 | c.2315A>G | p.Asn772Ser |
| NaV1.1 s | c.354G>C | p.Arg118Ser | NaV1.1 s | c.4351C>G | p.Pro1451Ala | NaV1.2 | c.2318C>T | p.Thr773Ile |
| NaV1.1 s | c.371T>A | p.Ile124Asn | NaV1.1 s | c.4351C>T | p.Pro1451Ser | NaV1.2 | c.2351C>T | p.Thr784Met |
| NaV1.1 s | c.379C>G | p.His127Asp | NaV1.1 s | c.4351C>A | p.Pro1451Thr | NaV1.2 | c.2435C>T | p.Ala812Val |
| NaV1.1 s | c.413T>C | p.Ile138Thr | NaV1.1 s | c.4352C>T | p.Pro1451Leu | NaV1.2 | c.2464G>A | p.Gly822Ser |
| NaV1.1 s | c.419C>G | p.Thr140Arg | NaV1.1 s | c.4358A>G | p.Tyr1453Cys | NaV1.2 | c.2483G>T | p.Gly828Val |
| NaV1.1 s | c.484A>C | p.Thr162Pro | NaV1.1 s | c.4360G>A | p.Glu1454Lys | NaV1.2 | c.2545C>A | p.Leu849Ile |
| NaV1.1 s | c.488G>A | p.Gly163Glu | NaV1.1 s | c.4381C>A | p.Leu1461Ile | NaV1.2 | c.2549G>C | p.Arg850Pro |
| NaV1.1 s | c.512T>G | p.Ile171Arg | NaV1.1 s | c.4384T>C | p.Tyr1462His | NaV1.2 | c.2558G>A | p.Arg853Gln |
| NaV1.1 s | c.512T>A | p.Ile171Lys | NaV1.1 s | c.4385A>G | p.Tyr1462Cys | NaV1.2 | c.2567G>A | p.Arg856Gln |
| NaV1.1 s | c.523G>A | p.Ala175Thr | NaV1.1 s | c.4387T>C | p.Phe1463Leu | NaV1.2 | c.2567G>T | p.Arg856Leu |
| NaV1.1 s | c.524C>T | p.Ala175Val | NaV1.1 s | c.4388T>C | p.Phe1463Ser | NaV1.2 | c.2588C>T | p.Ser863Phe |
| NaV1.1 s | c.529G>A | p.Gly177Arg | NaV1.1 s | c.4408G>T | p.Gly1470Trp | NaV1.2 | c.2619C>G | p.Ile873Met |
| NaV1.1 s | c.530G>A | p.Gly177Glu | NaV1.1 s | c.4415T>C | p.Phe1472Ser | NaV1.2 | c.2627A>C | p.Asn876Thr |
| NaV1.1 s | c.535T>C | p.Cys179Arg | NaV1.1 s | c.4424T>C | p.Leu1475Ser | NaV1.2 | c.2635G>A | p.Gly879Arg |
| NaV1.1 s | c.554T>C | p.Phe185Ser | NaV1.1 s | c.4428C>A | p.Asn1476Lys | NaV1.2 | c.2638G>T | p.Ala880Ser |
| NaV1.1 s | c.568T>C | p.Trp190Arg | NaV1.1 s | c.4451A>G | p.Asp1484Gly | NaV1.2 | c.2642T>C | p.Leu881Pro |
| NaV1.1 s | c.571A>T | p.Asn191Tyr | NaV1.1 s | c.4453A>G | p.Asn1485Asp | NaV1.2 | c.2644G>C | p.Gly882Arg |
| NaV1.1 s | c.572A>C | p.Asn191Thr | NaV1.1 s | c.4453A>T | p.Asn1485Tyr | NaV1.2 | c.2645G>A | p.Gly882Glu |
| NaV1.1 s | c.573C>G | p.Asn191Lys | NaV1.1 s | c.4467G>C | p.Gln1489His | NaV1.2 | c.2651T>A | p.Leu884His |
| NaV1.1 s | c.580G>A | p.Asp194Asn | NaV1.1 s | c.4490A>G | p.Asp1497Gly | NaV1.2 | c.2654C>T | p.Thr885Ile |
| NaV1.1 s | c.580G>C | p.Asp194His | NaV1.1 s | c.4507G>A | p.Glu1503Lys | NaV1.2 | c.2660T>C | p.Val887Ala |
| NaV1.1 s | c.580G>A | p.Asp194Asn | NaV1.1 s | c.4508A>G | p.Glu1503Gly | NaV1.2 | c.2670C>G | p.Ile890Met |
| NaV1.1 s | c.581A>C | p.Asp194Ala | NaV1.1 s | c.4519T>G | p.Tyr1507Asp | NaV1.2 | c.2672T>C | p.Ile891Thr |
| NaV1.1 s | c.581A>G | p.Asp194Gly | NaV1.1 s | c.4555C>A | p.Pro1519Thr | NaV1.2 | c.2674G>A | p.Val892Ile |
| NaV1.1 s | c.596C>G | p.Thr199Arg | NaV1.1 s | c.4568T>C | p.Ile1523Thr | NaV1.2 | c.2684T>C | p.Phe895Ser |
| NaV1.1 s | c.604T>G | p.Tyr202Asp | NaV1.1 s | c.4584C>G | p.Asn1528Lys | NaV1.2 | c.2695G>A | p.Gly899Ser |
| NaV1.1 s | c.629G>A | p.Gly210Asp | NaV1.1 s | c.4612G>A | p.Val1538Ile | NaV1.2 | c.2715G>C | p.Lys905Asn |
| NaV1.1 s | c.635T>C | p.Val212Ala | NaV1.1 s | c.4615C>A | p.Thr1539Pro | NaV1.2 | c.2722A>G | p.Lys908Glu |
| NaV1.1 s | c.650C>A | p.Thr217Lys | NaV1.1 s | c.4631A>C | p.Asp1544Ala | NaV1.2 | c.2783T>G | p.Phe928Cys |
| NaV1.1 s | c.668C>A | p.Ala223Glu | NaV1.1 s | c.4631A>G | p.Asp1544Gly | NaV1.2 | c.2790C>A | p.His930Gln |
| NaV1.1 s | c.675G>C | p.Lys225Asn | NaV1.1 s | c.4633A>G | p.Ile1545Val | NaV1.2 | c.2809C>T | p.Arg937Cys |
| NaV1.1 s | c.677C>A | p.Thr226Lys | NaV1.1 s | c.4634T>G | p.Ile1545Arg | NaV1.2 | c.2810G>A | p.Arg937His |
| NaV1.1 s | c.677C>T | p.Thr226Met | NaV1.1 s | c.4664T>G | p.Met1555Arg | NaV1.2 | c.2872A>G | p.Met958Val |
| NaV1.1 s | c.677C>G | p.Thr226Arg | NaV1.1 s | c.4681G>A | p.Glu1561Lys | NaV1.2 | c.2894T>G | p.Met965Arg |
| NaV1.1 s | c.680T>G | p.Ile227Ser | NaV1.1 s | c.4723C>T | p.Arg1575Cys | NaV1.2 | c.2928C>A | p.Asn976Lys |
| NaV1.1 s | c.680T>C | p.Ile227Thr | NaV1.1 s | c.4736T>A | p.Val1579Glu | NaV1.2 | c.2932T>C | p.Phe978Leu |
| NaV1.1 s | c.694G>A | p.Gly232Ser | NaV1.1 s | c.4757G>A | p.Gly1586Glu | NaV1.2 | c.2948T>G | p.Leu983Trp |
| NaV1.1 s | c.698T>G | p.Leu233Arg | NaV1.1 s | c.4762T>C | p.Cys1588Arg | NaV1.2 | c.2960G>T | p.Ser987Ile |
| NaV1.1 s | c.715G>A | p.Ala239Thr | NaV1.1 s | c.4775T>A | p.Leu1592His | NaV1.2 | c.2989G>T | p.Asp997Tyr |
| NaV1.1 s | c.716C>T | p.Ala239Val | NaV1.1 s | c.4775T>C | p.Leu1592Pro | NaV1.2 | c.2995G>A | p.Glu999Lys |
| NaV1.1 s | c.719T>C | p.Leu240Pro | NaV1.1 s | c.4786C>T | p.Arg1596Cys | NaV1.2 | c.2996A>T | p.Glu999Val |
| NaV1.1 s | c.728C>T | p.Ser243Phe | NaV1.1 s | c.4787G>T | p.Arg1596Leu | NaV1.2 | c.3003T>A | p.Asn1001Lys |
| NaV1.1 s | c.728C>A | p.Ser243Tyr | NaV1.1 s | c.4814A>G | p.Asn1605Ser | NaV1.2 | c.3007C>A | p.Leu1003Ile |
| NaV1.1 s | c.755T>A | p.Ile252Asn | NaV1.1 s | c.4822G>T | p.Asp1608Tyr | NaV1.2 | c.3149A>T | p.Asp1050Val |
| NaV1.1 s | c.769T>C | p.Cys257Arg | NaV1.1 s | c.4823A>G | p.Asp1608Gly | NaV1.2 | c.3342T>A | p.Phe1114Leu |
| NaV1.1 s | c.777C>A | p.Ser259Arg | NaV1.1 s | c.4834G>A | p.Val1612Ile | NaV1.2 | c.3343G>A | p.Glu1115Lys |
| NaV1.1 s | c.793G>T | p.Gly265Trp | NaV1.1 s | c.4888G>C | p.Val1630Leu | NaV1.2 | c.3370A>T | p.Ser1124Cys |
| NaV1.1 s | c.829T>C | p.Cys277Arg | NaV1.1 s | c.4888G>A | p.Val1630Met | NaV1.2 | c.3383T>C | p.Met1128Thr |
| NaV1.1 s | c.829T>G | p.Cys277Gly | NaV1.1 s | c.4894C>T | p.Pro1632Ser | NaV1.2 | c.3524G>A | p.Cys1175Tyr |
| NaV1.1 s | c.838T>C | p.Trp280Arg | NaV1.1 s | c.4910T>A | p.Val1637Glu | NaV1.2 | c.3631G>A | p.Glu1211Lys |
| NaV1.1 s | c.840G>T | p.Trp280Cys | NaV1.1 s | c.4913T>C | p.Ile1638Thr | NaV1.2 | c.3667G>C | p.Gly1223Arg |
| NaV1.1 s | c.841C>T | p.Pro281Ser | NaV1.1 s | c.4913T>A | p.Ile1638Asn | NaV1.2 | c.3778A>G | p.Lys1260Glu |
| NaV1.1 s | c.841C>G | p.Pro281Ala | NaV1.1 s | c.4915C>G | p.Arg1639Gly | NaV1.2 | c.3778A>C | p.Lys1260Gln |
| NaV1.1 s | c.842C>T | p.Pro281Leu | NaV1.1 s | c.4925G>T | p.Arg1642Met | NaV1.2 | c.3841A>T | p.Ile1281Phe |
| NaV1.1 s | c.866A>T | p.Glu289Val | NaV1.1 s | c.4926G>C | p.Arg1642Ser | NaV1.2 | c.3844G>T | p.Val1282Phe |
| NaV1.1 s | c.890C>T | p.Thr297Ile | NaV1.1 s | c.4934G>A | p.Arg1645Gln | NaV1.2 | c.3928G>T | p.Ala1310Ser |
| NaV1.1 s | c.935T>C | p.Phe312Ser | NaV1.1 s | c.4942C>T | p.Arg1648Cys | NaV1.2 | c.3935G>C | p.Arg1312Thr |
| NaV1.1 s | c.949T>C | p.Tyr317His | NaV1.1 s | c.4943G>A | p.Arg1648His | NaV1.2 | c.3955C>T | p.Arg1319Trp |
| NaV1.1 s | c.965G>T | p.Arg322Ile | NaV1.1 s | c.4958C>A | p.Ala1653Glu | NaV1.2 | c.3956G>C | p.Arg1319Pro |
| NaV1.1 s | c.970C>A | p.His324Asn | NaV1.1 s | c.4969C>T | p.Arg1657Cys | NaV1.2 | c.3956G>T | p.Arg1319Leu |
| NaV1.1 s | c.974A>G | p.Tyr325Cys | NaV1.1 s | c.4973C>G | p.Thr1658Arg | NaV1.2 | c.3956G>A | p.Arg1319Gln |
| NaV1.1 s | c.1007G>A | p.Cys336Tyr | NaV1.1 s | c.4973C>T | p.Thr1658Met | NaV1.2 | c.3961G>A | p.Glu1321Lys |
| NaV1.1 s | c.1010G>A | p.Gly337Glu | NaV1.1 s | c.4979T>C | p.Leu1660Pro | NaV1.2 | c.3967A>G | p.Met1323Val |
| NaV1.1 s | c.1019C>T | p.Ser340Phe | NaV1.1 s | c.4982T>C | p.Phe1661Ser | NaV1.2 | c.3973G>T | p.Val1325Phe |
| NaV1.1 s | c.1025C>T | p.Ala342Val | NaV1.1 s | c.4985C>T | p.Ala1662Val | NaV1.2 | c.3976G>C | p.Val1326Leu |
| NaV1.1 s | c.1027G>C | p.Gly343Arg | NaV1.1 s | c.4991T>A | p.Met1664Lys | NaV1.2 | c.3977T>A | p.Val1326Asp |
| NaV1.1 s | c.1027G>T | p.Gly343Cys | NaV1.1 s | c.5000T>C | p.Leu1667Pro | NaV1.2 | c.3988C>T | p.Leu1330Phe |
| NaV1.1 s | c.1028G>A | p.Gly343Asp | NaV1.1 s | c.5002C>G | p.Pro1668Ala | NaV1.2 | c.4007C>A | p.Ser1336Tyr |
| NaV1.1 s | c.1033T>C | p.Cys345Arg | NaV1.1 s | c.5003C>G | p.Pro1668Arg | NaV1.2 | c.4013T>C | p.Met1338Thr |
| NaV1.1 s | c.1034G>A | p.Cys345Tyr | NaV1.1 s | c.5003C>T | p.Pro1668Leu | NaV1.2 | c.4015A>G | p.Asn1339Asp |
| NaV1.1 s | c.1035T>G | p.Cys345Trp | NaV1.1 s | c.5014A>C | p.Asn1672His | NaV1.2 | c.4022T>G | p.Leu1341Arg |
| NaV1.1 s | c.1046A>G | p.Tyr349Cys | NaV1.1 s | c.5015A>T | p.Asn1672Ile | NaV1.2 | c.4025T>C | p.Leu1342Pro |
| NaV1.1 s | c.1053T>G | p.Cys351Trp | NaV1.1 s | c.5018T>C | p.Ile1673Thr | NaV1.2 | c.4156T>C | p.Cys1386Arg |
| NaV1.1 s | c.1064G>A | p.Gly355Asp | NaV1.1 s | c.5020G>C | p.Gly1674Arg | NaV1.2 | c.4223T>C | p.Val1408Ala |
| NaV1.1 s | c.1066A>G | p.Arg356Gly | NaV1.1 s | c.5027T>A | p.Leu1676Gln | NaV1.2 | c.4259C>T | p.Thr1420Met |
| NaV1.1 s | c.1070A>T | p.Asn357Ile | NaV1.1 s | c.5029C>T | p.Leu1677Phe | NaV1.2 | c.4264A>G | p.Lys1422Glu |
| NaV1.1 s | c.1072C>A | p.Pro358Thr | NaV1.1 s | c.5048T>C | p.Ile1683Thr | NaV1.2 | c.4378G>C | p.Gly1460Arg |
| NaV1.1 s | c.1076A>G | p.Asn359Ser | NaV1.1 s | c.5050T>G | p.Tyr1684Asp | NaV1.2 | c.4405A>T | p.Ile1469Phe |
| NaV1.1 s | c.1087A>C | p.Thr363Pro | NaV1.1 s | c.5051A>C | p.Tyr1684Ser | NaV1.2 | c.4419A>G | p.Ile1473Met |
| NaV1.1 s | c.1088C>G | p.Thr363Arg | NaV1.1 s | c.5054C>A | p.Ala1685Asp | NaV1.2 | c.4436A>C | p.Gln1479Pro |
| NaV1.1 s | c.1094T>C | p.Phe365Ser | NaV1.1 s | c.5062G>T | p.Gly1688Trp | NaV1.2 | c.4463T>A | p.Ile1488Asn |
| NaV1.1 s | c.1098T>A | p.Asp366Glu | NaV1.1 s | c.5066T>G | p.Met1689Arg | NaV1.2 | c.4471A>G | p.Thr1491Ala |
| NaV1.1 s | c.1125G>T | p.Leu375Phe | NaV1.1 s | c.5075T>C | p.Phe1692Ser | NaV1.2 | c.4498G>A | p.Ala1500Thr |
| NaV1.1 s | c.1130G>T | p.Arg377Leu | NaV1.1 s | c.5081A>G | p.Tyr1694Cys | NaV1.2 | c.4565G>C | p.Gly1522Ala |
| NaV1.1 s | c.1130G>A | p.Arg377Gln | NaV1.1 s | c.5106T>A | p.Asp1702Glu | NaV1.2 | c.4591C>A | p.Gln1531Lys |
| NaV1.1 s | c.1133T>A | p.Leu378Gln | NaV1.1 s | c.5107G>T | p.Asp1703Tyr | NaV1.2 | c.4606A>C | p.Ser1536Arg |
| NaV1.1 s | c.1136T>G | p.Met379Arg | NaV1.1 s | c.5108A>T | p.Asp1703Val | NaV1.2 | c.4633A>G | p.Met1545Val |
| NaV1.1 s | c.1144G>A | p.Asp382Asn | NaV1.1 s | c.5119T>G | p.Phe1707Val | NaV1.2 | c.4642A>G | p.Met1548Val |
| NaV1.1 s | c.1149C>G | p.Phe383Asp | NaV1.1 s | c.5126C>T | p.Thr1709Ile | NaV1.2 | c.4687C>G | p.Leu1563Val |
| NaV1.1 s | c.1150T>C | p.Trp384Arg | NaV1.1 s | c.5136C>G | p.Asn1712Lys | NaV1.2 | c.4712T>C | p.Ile1571Thr |
| NaV1.1 s | c.1154A>G | p.Glu385Gly | NaV1.1 s | c.5138G>A | p.Ser1713Asn | NaV1.2 | c.4726G>A | p.Gly1576Arg |
| NaV1.1 s | c.1162T>C | p.Tyr388His | NaV1.1 s | c.5141T>G | p.Met1714Arg | NaV1.2 | c.4751C>G | p.Ser1584Cys |
| NaV1.1 s | c.1171A>C | p.Thr391Pro | NaV1.1 s | c.5141T>A | p.Met1714Lys | NaV1.2 | c.4766A>G | p.Tyr1589Cys |
| NaV1.1 s | c.1176A>T | p.Leu392Phe | NaV1.1 s | c.5146T>C | p.Cys1716Arg | NaV1.2 | c.4777G>A | p.Gly1593Arg |
| NaV1.1 s | c.1177C>T | p.Arg393Cys | NaV1.1 s | c.5150T>C | p.Leu1717Pro | NaV1.2 | c.4780T>A | p.Trp1594Arg |
| NaV1.1 s | c.1177C>A | p.Arg393Ser | NaV1.1 s | c.5162C>G | p.Thr1721Arg | NaV1.2 | c.4787T>G | p.Ile1596Ser |
| NaV1.1 s | c.1178G>A | p.Arg393His | NaV1.1 s | c.5170G>A | p.Ala1724Thr | NaV1.2 | c.4789T>C | p.Phe1597Leu |
| NaV1.1 s | c.1187G>A | p.Gly396Glu | NaV1.1 s | c.5173G>T | p.Gly1725Cys | NaV1.2 | c.4793A>G | p.Asp1598Gly |
| NaV1.1 s | c.1207T>C | p.Phe403Leu | NaV1.1 s | c.5176T>C | p.Trp1726Arg | NaV1.2 | c.4793A>T | p.Asp1598Val |
| NaV1.1 s | c.1207T>G | p.Phe403Val | NaV1.1 s | c.5180A>G | p.Asp1727Gly | NaV1.2 | c.4835C>G | p.Ala1612Gly |
| NaV1.1 s | c.1208T>C | p.Phe403Ser | NaV1.1 s | c.5186T>G | p.Leu1729Trp | NaV1.2 | c.4864C>T | p.Pro1622Ser |
| NaV1.1 s | c.1216G>T | p.Val406Phe | NaV1.1 s | c.5191G>C | p.Ala1731Pro | NaV1.2 | c.4868C>A | p.Thr1623Asn |
| NaV1.1 s | c.1226T>G | p.Leu409Trp | NaV1.1 s | c.5197A>G | p.Asn1733Asp | NaV1.2 | c.4877G>A | p.Arg1626Gln |
| NaV1.1 s | c.1234T>A | p.Phe412Ile | NaV1.1 s | c.5221T>C | p.Cys1741Arg | NaV1.2 | c.4879G>A | p.Val1627Met |
| NaV1.1 s | c.1237T>A | p.Tyr413Asn | NaV1.1 s | c.5246G>A | p.Gly1749Glu | NaV1.2 | c.4886G>A | p.Arg1629His |
| NaV1.1 s | c.1246A>T | p.Asn416Tyr | NaV1.1 s | c.5260G>A | p.Gly1754Arg | NaV1.2 | c.4886G>T | p.Arg1629Leu |
| NaV1.1 s | c.1264G>A | p.Val422Met | NaV1.1 s | c.5264 A>G | p.Asp1755Gly | NaV1.2 | c.4895G>A | p.Arg1632Lys |
| NaV1.1 s | c.1276T>A | p.Tyr426Asn | NaV1.1 s | c.5266T>C | p.Cys1756Arg | NaV1.2 | c.4901G>A | p.Gly1634Asp |
| NaV1.1 s | c.1277A>G | p.Tyr426Cys | NaV1.1 s | c.5266T>G | p.Cys1756Gly | NaV1.2 | c.4901G>T | p.Gly1634Val |
| NaV1.1 s | c.1574C>T | p.Ser525Phe | NaV1.1 s | c.5285G>A | p.Gly1762Glu | NaV1.2 | c.4904G>A | p.Arg1635Gln |
| NaV1.1 s | c.1625G>A | p.Arg542Gln | NaV1.1 s | c.5287A>T | p.Ile1763Phe | NaV1.2 | c.4908C>G | p.Ile1636Met |
| NaV1.1 s | c.1662G>T | p.Gln554His | NaV1.1 s | c.5288T>A | p.Ile1763Asn | NaV1.2 | c.4918A>T | p.Ile1640Phe |
| NaV1.1 s | c.1811G>A | p.Arg604His | NaV1.1 s | c.5304T>G | p.Ser1768Arg | NaV1.2 | c.4919T>G | p.Ile1640Ser |
| NaV1.1 s | c.1876A>G | p.Ser626Gly | NaV1.1 s | c.5308A>T | p.Ile1770Phe | NaV1.2 | c.4923A>C | p.Lys1641Asn |
| NaV1.1 s | c.2021A>G | p.Asp674Gly | NaV1.1 s | c.5309T>A | p.Ile1770Asn | NaV1.2 | c.4949T>C | p.Leu1650Pro |
| NaV1.1 s | c.2092C>T | p.His698Tyr | NaV1.1 s | c.5309T>C | p.Ile1770Thr | NaV1.2 | c.4952T>G | p.Phe1651Cys |
| NaV1.1 s | c.2213G>T | p.Trp738Leu | NaV1.1 s | c.5312T>A | p.Ile1771Asn | NaV1.2 | c.4954G>C | p.Ala1652Pro |
| NaV1.1 s | c.2284A>G | p.Asn762Asp | NaV1.1 s | c.5318C>T | p.Ser1773Phe | NaV1.2 | c.4967C>T | p.Ser1656Phe |
| NaV1.1 s | c.2303C>T | p.Pro768Leu | NaV1.1 s | c.5326G>T | p.Val1776Phe | NaV1.2 | c.4979T>G | p.Leu1660Trp |
| NaV1.1 s | c.2348T>C | p.Leu783Pro | NaV1.1 s | c.5339T>C | p.Met1780Thr | NaV1.2 | c.4993C>T | p.Leu1665Phe |
| NaV1.1 s | c.2354T>C | p.Met785Thr | NaV1.1 s | c.5341T>C | p.Tyr1781His | NaV1.2 | c.5045T>C | p.Phe1682Ser |
| NaV1.1 s | c.2362G>A | p.Glu788Lys | NaV1.1 s | c.5342A>G | p.Tyr1781Cys | NaV1.2 | c.5192G>A | p.Cys1731Tyr |
| NaV1.1 s | c.2378C>T | p.Thr793Met | NaV1.1 s | c.5345T>G | p.Ile1782Ser | NaV1.2 | c.5231G>A | p.Gly1744Glu |
| NaV1.1 s | c.2435C>G | p.Thr812Arg | NaV1.1 s | c.5346C>G | p.Ile1782Met | NaV1.2 | c.5230G>A | p.Gly1744Arg |
| NaV1.1 s | c.2435C>T | p.Thr812Ile | NaV1.1 s | c.5347G>A | p.Ala1783Thr | NaV1.2 | c.5272A>C | p.Ser1758Arg |
| NaV1.1 s | c.2525T>G | p.Leu842Arg | NaV1.1 s | c.5347G>A | p.Ala1783Thr | NaV1.2 | c.5294T>C | p.Leu1765Pro |
| NaV1.1 s | c.2527A>C | p.Phe843Arg | NaV1.1 s | c.5348C>T | p.Ala1783Val | NaV1.2 | c.5317G>A | p.Ala1773Thr |
| NaV1.1 s | c.2529C>G | p.Ser843Arg | NaV1.1 s | c.5359G>A | p.Glu1787Lys | NaV1.2 | c.5318C>T | p.Ala1773Val |
| NaV1.1 s | c.2536G>A | p.Glu853Lys | NaV1.1 s | c.5374G>A | p.Ala1792Thr | NaV1.2 | c.5333A>T | p.Asn1778Ile |
| NaV1.1 s | c.2537A>G | p.Glu846Gly | NaV1.1 s | c.5375C>G | p.Ala1792Gly | NaV1.2 | c.5339G>T | p.Ser1780Ile |
| NaV1.1 s | c.2557G>A | p.Glu853Lys | NaV1.1 s | c.5422T>A | p.Phe1808Ile | NaV1.2 | c.5431C>G | p.Gln1811Glu |
| NaV1.1 s | c.2575C>T | p.Arg859Cys | NaV1.1 s | c.5422T>C | p.Phe1808Leu | NaV1.2 | c.5485C>T | p.Leu1829Phe |
| NaV1.1 s | c.2585G>A | p.Arg862Gln | NaV1.1 s | c.5434T>G | p.Trp1812Gly | NaV1.2 | c.5558A>G | p.His1853Arg |
| NaV1.1 s | c.2588T>G | p.Leu863Trp | NaV1.1 s | c.5435G>C | p.Trp1812Ser | NaV1.2 | c.5616G>A | p.Met1872Ile |
| NaV1.1 s | c.2593C>G | p.Arg865Gly | NaV1.1 s | c.5468T>G | p.Met1823Arg | NaV1.2 | c.5623C>T | p.Leu1875Phe |
| NaV1.1 s | c.2615C>A | p.Ser872Tyr | NaV1.1 s | c.5474T>C | p.Phe1825Ser | NaV1.2 | c.5638G>A | p.Glu1880Lys |
| NaV1.1 s | c.2624C>A | p.Thr875Lys | NaV1.1 s | c.5492T>C | p.Phe1831Ser | NaV1.2 | c.5644C>G | p.Arg1882Gly |
| NaV1.1 s | c.2624C>T | p.Thr875Met | NaV1.1 s | c.5494G>C | p.Ala1832Pro | NaV1.2 | c.5645G>A | p.Arg1882Gln |
| NaV1.1 s | c.2626T>A | p.Leu876Ile | NaV1.1 s | c.5503C>T | p.Leu1835Phe | NaV1.2 | c.5645G>C | p.Arg1882Pro |
| NaV1.1 s | c.2644A>T | p.Ile882Phe | NaV1.1 s | c.5515C>G | p.Leu1839Val | NaV1.2 | c.5645G>T | p.Arg1882Leu |
| NaV1.1 s | c.2669T>C | p.Leu890Phe | NaV1.1 s | c.5534A>C | p.Asn1845Thr | NaV1.2 | c.5704C>T | p.Arg1902Cys |
| NaV1.1 s | c.2686G>A | p.Val896Ile | NaV1.1 s | c.5555T>A | p.Met1852Lys | NaV1.2 | c.5710C>G | p.Gln1904Glu |
| NaV1.1 s | c.2686G>C | p.Val896Leu | NaV1.1 s | c.5555T>C | p.Met1852Thr | NaV1.2 | c.5753G>A | p.Arg1918His |
| NaV1.1 s | c.2686G>T | p.Val896Phe | NaV1.1 s | c.5564C>T | p.Pro1855Leu | NaV1.2 | c.5798A>T | p.Lys1933Met |
| NaV1.1 s | c.2705T>G | p.Phe902Cys | NaV1.1 s | c.5597A>G | p.Asp1866Gly | NaV1.2 | c.5921C>T | p.Ser1974Leu |
| NaV1.1 s | c.2713G>A | p.Ala905Thr | NaV1.1 s | c.5627T>C | p.Leu1876Pro | NaV1.2 | c.5995G>T | p.Asp1999Tyr |
| NaV1.1 s | c.2728C>A | p.Gln910Lys | NaV1.1 s | c.5639G>A | p.Gly1880Glu | NaV1.6 | c.134G>A | p.Arg45Gln |
| NaV1.1 s | c.2729A>T | p.Gln910Leu | NaV1.1 s | c.5641G>A | p.Glu1881Lys | NaV1.6 | c.172G>A | p.Asp58Asn |
| NaV1.1 s | c.2780G>T | p.Cys927Phe | NaV1.1 s | c.5643G>C | p.Glu1881Asp | NaV1.6 | c.302A>G | p.Lys101Arg |
| NaV1.1 s | c.2791C>T | p.Arg931Cys | NaV1.1 s | c.5657G>A | p.Arg1886Gln | NaV1.6 | c.424A>G | p.Ile142Val |
| NaV1.1 s | c.2792G>A | p.Arg931His | NaV1.1 s | c.5726C>T | p.Thr1909Ile | NaV1.6 | c.497C>T | p.Thr166Ile |
| NaV1.1 s | c.2792G>C | p.Arg931Pro | NaV1.1 s | c.5765T>C | p.Ile1922Thr | NaV1.6 | c.599G>A | p.Ser200Asn |
| NaV1.1 s | c.2796G>C | p.Trp932Cys | NaV1.1 s | c.5782C>G | p.Arg1928Gly | NaV1.6 | c.628T>C | p.Phe210Leu |
| NaV1.1 s | c.2798A>C | p.His933Pro | NaV1.1 m | c.80G>C | p.Arg27Thr | NaV1.6 | c.632T>C | p.Val211Ala |
| NaV1.1 s | c.2802G>C | p.Met934Ile | NaV1.1 m | c.82C>T | p.Arg28Cys | NaV1.6 | c.631G>C | p.Val211Leu |
| NaV1.1 s | c.2807A>T | p.Asp936Val | NaV1.1 m | c.133G>A | p.Asp45Asn | NaV1.6 | c.641G>A | p.Gly214Asp |
| NaV1.1 s | c.2815C>T | p.His939Tyr | NaV1.1 m | c.220T>C | p.Ser74Pro | NaV1.6 | c.643A>G | p.Asn215Asp |
| NaV1.1 s | c.2816A>G | p.His939Arg | NaV1.1 m | c.307A>G | p.Ser103Gly | NaV1.6 | c.647T>A | p.Val216Asp |
| NaV1.1 s | c.2817C>G | p.His939Gln | NaV1.1 m | c.323T>G | p.Leu108Arg | NaV1.6 | c.667A>G | p.Arg223Gly |
| NaV1.1 s | c.2819C>T | p.Ser940Phe | NaV1.1 m | c.434T>C | p.Met145Thr | NaV1.6 | c.669G>C | p.Arg223Ser |
| NaV1.1 s | c.2825T>C | p.Leu942Pro | NaV1.1 m | c.563A>T | p.Asp188Val | NaV1.6 | c.694T>C | p.Ser232Pro |
| NaV1.1 s | c.2828T>A | p.Ile943Asp | NaV1.1 m | c.563A>T | p.Asp188Val | NaV1.6 | c.697G>A | p.Val233Ile |
| NaV1.1 s | c.2831T>A | p.Val944Glu | NaV1.1 m | c.577C>T | p.Leu193Phe | NaV1.6 | c.715A>G | p.Thr239Ala |
| NaV1.1 s | c.2831T>C | p.Val944Ala | NaV1.1 m | c.650C>G | p.Thr217Arg | NaV1.6 | c.718A>C | p.Ile240Leu |
| NaV1.1 s | c.2833T>C | p.Phe945Leu | NaV1.1 m | c.652T>C | p.Phe218Leu | NaV1.6 | c.718A>G | p.Ile240Val |
| NaV1.1 s | c.2836C>T | p.Arg946Cys | NaV1.1 m | c.652T>C | p.Phe218Leu | NaV1.6 | c.769C>G | p.Leu257Val |
| NaV1.1 s | c.2837G>A | p.Arg946His | NaV1.1 m | c.677C>T | p.Thr226Met | NaV1.6 | c.779T>C | p.Phe260Ser |
| NaV1.1 s | c.2837G>C | p.Arg946Pro | NaV1.1 m | c.825T>A | p.Asn275Lys | NaV1.6 | c.782G>T | p.Cys261Phe |
| NaV1.1 s | c.2837G>A | p.Arg946His | NaV1.1 m | c.871A>G | p.Ser291Gly | NaV1.6 | c.800T>C | p.Leu267Ser |
| NaV1.1 s | c.2846G>C | p.Cys949Ser | NaV1.1 m | c.1144G>A | p.Asp382Asn | NaV1.6 | c.802A>C | p.Ile268Leu |
| NaV1.1 s | c.2846G>A | p.Cys949Tyr | NaV1.1 m | c.1183G>C | p.Ala395Pro | NaV1.6 | c.971G>C | p.Cys324Tyr |
| NaV1.1 s | c.2848G>C | p.Gly950Arg | NaV1.1 m | c.1222T>C | p.Phe408Leu | NaV1.6 | c.1099A>G | p.Met367Val |
| NaV1.1 s | c.2849G>A | p.Gly950Glu | NaV1.1 m | c.1234T>A | p.Phe412Ile | NaV1.6 | c.1142G>A | p.Arg381Gln |
| NaV1.1 s | c.2854T>C | p.Trp952Arg | NaV1.1 m | c.1265T>A | p.Val422Glu | NaV1.6 | c.1150G>A | p.Gly384Arg |
| NaV1.1 s | c.2854T>G | p.Trp952Gly | NaV1.1 m | c.1625G>A | p.Arg542Gln | NaV1.6 | c.1157C>G | p.Thr386Arg |
| NaV1.1 s | c.2860G>A | p.Glu954Lys | NaV1.1 m | c.1876A>G | p.Ser626Gly | NaV1.6 | c.1195T>C | p.Ser399Pro |
| NaV1.1 s | c.2864C>T | p.Thr955Ile | NaV1.1 m | c.2369A>G | p.Tyr790Cys | NaV1.6 | c.1221G>C | p.Leu407Phe |
| NaV1.1 s | c.2867T>A | p.Met956Lys | NaV1.1 m | c.2369A>G | p.Tyr790Cys | NaV1.6 | c.1222G>A | p.Ala408Thr |
| NaV1.1 s | c.2869T>C | p.Trp957Arg | NaV1.1 m | c.2369A>T | p.Tyr790Phe | NaV1.6 | c.1228G>C | p.Val410Leu |
| NaV1.1 s | c.2870G>T | p.Trp957Leu | NaV1.1 m | c.2576G>A | p.Arg859His | NaV1.6 | c.1244A>G | p.Glu415Gly |
| NaV1.1 s | c.2875T>C | p.Cys959Arg | NaV1.1 m | c.2576G>A | p.Arg859His | NaV1.6 | c.1246G>A | p.Glu416Lys |
| NaV1.1 s | c.2876G>A | p.Cys959Tyr | NaV1.1 m | c.2803A>C | p.Asn935His | NaV1.6 | c.1250A>C | p.Gln417Pro |
| NaV1.1 s | c.2878A>G | p.Met960Val | NaV1.1 m | c.2879T>C | p.Met960Thr | NaV1.6 | c.1588C>T | p.Arg530Trp |
| NaV1.1 s | c.2918T>A | p.Met973Lys | NaV1.1 m | c.2901G>T | p.Met967Ile | NaV1.6 | c.1589G>A | p.Arg530Gln |
| NaV1.1 s | c.2928G>A | p.Met976Ile | NaV1.1 m | c.2917A>G | p.Met973Val | NaV1.6 | c.1792C>T | p.Arg598Trp |
| NaV1.1 s | c.2935G>A | p.Gly979Arg | NaV1.1 m | c.2927T>C | p.Met976Thr | NaV1.6 | c.1819G>A | p.Ala607Thr |
| NaV1.1 s | c.2936G>T | p.Gly979Val | NaV1.1 m | c.2928G>A | p.Met976Ile | NaV1.6 | c.1984C>T | p.Arg662Cys |
| NaV1.1 s | c.2942T>C | p.Leu981Pro | NaV1.1 m | c.3241G>A | p.Gly1081Arg | NaV1.6 | c.2074G>A | p.Gly692Arg |
| NaV1.1 s | c.2944G>C | p.Val982Leu | NaV1.1 m | c.3521C>G | p.Thr1174Ser | NaV1.6 | c.2287A>G | p.Ile763Val |
| NaV1.1 s | c.2947G>T | p.Val983Phe | NaV1.1 m | c.3610T>C | p.Trp1204Arg | NaV1.6 | c.2300C>T | p.Thr767Ile |
| NaV1.1 s | c.2948T>C | p.Val983Ala | NaV1.1 m | c.3610T>C | p.Trp1204Arg | NaV1.6 | c.2415A>G | p.Ile805Met |
| NaV1.1 s | c.2954A>T | p.Asn985Ile | NaV1.1 m | c.3611G>C | p.Trp1204Ser | NaV1.6 | c.2518C>T | p.Leu840Phe |
| NaV1.1 s | c.2956C>T | p.Leu986Phe | NaV1.1 m | c.3688C>T | p.Leu1230Phe | NaV1.6 | c.2519T>C | p.Leu840Pro |
| NaV1.1 s | c.2959T>C | p.Phe987Leu | NaV1.1 m | c.3748G>C | p.Lys1249Asn | NaV1.6 | c.2534C>T | p.Ser845Phe |
| NaV1.1 s | c.2970G>T | p.Leu990Phe | NaV1.1 m | c.3749C>T | p.Thr1250Met | NaV1.6 | c.2533T>C | p.Ser845Pro |
| NaV1.1 s | c.2979C>G | p.Ser993Arg | NaV1.1 m | c.3809A>C | p.Lys1270Thr | NaV1.6 | c.2537T>C | p.Phe846Ser |
| NaV1.1 s | c.2983T>C | p.Phe995Leu | NaV1.1 m | c.3820T>A | p.Tyr1274Asn | NaV1.6 | c.2549G>A | p.Arg850Gln |
| NaV1.1 s | c.2993A>G | p.Asp998Gly | NaV1.1 m | c.3820T>A | p.Tyr1274Asn | NaV1.6 | c.2603C>T | p.Ile868Thr |
| NaV1.1 s | c.3044C>T | p.Ala1015Val | NaV1.1 m | c.3924A>T | p.Glu1308Asp | NaV1.6 | c.2620G>A | p.Ala874Thr |
| NaV1.1 s | c.3166A>G | p.Asn1056Asp | NaV1.1 m | c.3925C>T | p.Leu1309Phe | NaV1.6 | c.2624T>A | p.Leu875Gln |
| NaV1.1 s | c.3196A>G | p.Thr1066Ala | NaV1.1 m | c.3967C>T | p.Pro1323Ser | NaV1.6 | c.2642T>C | p.Val881Ala |
| NaV1.1 s | c.3202G>A | p.Glu1068Lys | NaV1.1 m | c.3968C>G | p.Pro1323Arg | NaV1.6 | c.2668G>A | p.Ala890Thr |
| NaV1.1 s | c.3521C>G | p.Thr1174Ser | NaV1.1 m | c.3977C>T | p.Ala1326Val | NaV1.6 | c.2671G>A | p.Val891Met |
| NaV1.1 s | c.3620T>C | p.Leu1207Pro | NaV1.1 m | c.3998T>C | p.Met1333Thr | NaV1.6 | c.2879T>A | p.Val960Asp |
| NaV1.1 s | c.3623G>A | p.Arg1208Lys | NaV1.1 m | c.4057G>C | p.Val1353Leu | NaV1.6 | c.2890G>C | p.Gly964Arg |
| NaV1.1 s | c.3629C>A | p.Thr1210Lys | NaV1.1 m | c.4096G>A | p.Val1366Ile | NaV1.6 | c.2905C>A | p.Leu969Met |
| NaV1.1 s | c.3641T>G | p.Ile1214Arg | NaV1.1 m | c.4096G>A | p.Val1366Ile | NaV1.6 | c.2932A>G | p.Ser978Gly |
| NaV1.1 s | c.3646G>A | p.Glu1216Lys | NaV1.1 m | c.4114A>G | p.Lys1372Glu | NaV1.6 | c.2936C>T | p.Ser979Phe |
| NaV1.1 s | c.3661G>A | p.Glu1221Lys | NaV1.1 m | c.4135G>T | p.Val1379Leu | NaV1.6 | c.2952C>G | p.Asn984Lys |
| NaV1.1 s | c.3688C>T | p.Leu1230Phe | NaV1.1 m | c.4144G>C | p.Gly1382Arg | NaV1.6 | c.3148G>A | p.Gly1050Ser |
| NaV1.1 s | c.3692G>C | p.Ser1231Thr | NaV1.1 m | c.4240A>G | p.Asn1414Asp | NaV1.6 | c.3601G>A | p.Glu1201Lys |
| NaV1.1 s | c.3693T>A | p.Ser1231Arg | NaV1.1 m | c.4283T>C | p.Val1428Ala | NaV1.6 | c.3722A>G | p.Tyr1241Cys |
| NaV1.1 s | c.3697G>C | p.Gly1233Arg | NaV1.1 m | c.4314G>A | p.Met1438Ile | NaV1.6 | c.3922T>C | p.Ser1308Pro |
| NaV1.1 s | c.3706G>C | p.Ala1236Pro | NaV1.1 m | c.4349A>C | p.Gln1450Pro | NaV1.6 | c.3943G>A | p.Val1315Met |
| NaV1.1 s | c.3714A>C | p.Glu1238Asp | NaV1.1 m | c.4541T>C | p.Leu1514Ser | NaV1.6 | c.3960G>T | p.Leu1320Phe |
| NaV1.1 s | c.3715G>T | p.Asp1239Tyr | NaV1.1 m | c.4628T>C | p.Phe1543Ser | NaV1.6 | c.3967G>C | p.Ala1323Pro |
| NaV1.1 s | c.3716A>G | p.Asp1239Gly | NaV1.1 m | c.4748A>G | p.His1583Arg | NaV1.6 | c.3967G>T | p.Ala1323Ser |
| NaV1.1 s | c.3721T>C | p.Tyr1241His | NaV1.1 m | c.4787G>A | p.Arg1596His | NaV1.6 | c.3979A>G | p.Ile1327Val |
| NaV1.1 s | c.3734G>A | p.Arg1245Gln | NaV1.1 m | c.4787G>A | p.Arg1596His | NaV1.6 | c.3983T>C | p.Met1328Thr |
| NaV1.1 s | c.3749C>T | p.Thr1250Met | NaV1.1 m | c.4787G>A | p.Arg1596His | NaV1.6 | c.3985A>G | p.Asn1329Asp |
| NaV1.1 s | c.3764C>A | p.Ala1255Asp | NaV1.1 m | c.4793A>T | p.Tyr1598Phe | NaV1.6 | c.3991C>G | p.Leu1331Val |
| NaV1.1 s | c.3776T>G | p.Phe1259Cys | NaV1.1 m | c.4831G>T | p.Val1611Phe | NaV1.6 | c.3995T>G | p.Leu1332Arg |
| NaV1.1 s | c.3778A>C | p.Thr1260Pro | NaV1.1 m | c.4847T>C | p.Ile1616Thr | NaV1.6 | c.4214C>A | p.Ala1405Asp |
| NaV1.1 s | c.3789C>G | p.Phe1263Leu | NaV1.1 m | c.4847T>C | p.Ile1616Thr | NaV1.6 | c.4351G>A | p.Gly1451Ser |
| NaV1.1 s | c.3794T>C | p.Leu1265Pro | NaV1.1 m | c.4943G>A | p.Arg1648His | NaV1.6 | c.4382G>T | p.Gly1461Val |
| NaV1.1 s | c.3797A>C | p.Glu1266Ala | NaV1.1 m | c.4968C>G | p.Ile1656Met | NaV1.6 | c.4394A>T | p.Asp1465Val |
| NaV1.1 s | c.3824G>T | p.Gly1275Val | NaV1.1 m | c.4970G>A | p.Arg1657His | NaV1.6 | c.4397A>C | p.Asn1466Thr |
| NaV1.1 s | c.3851G>C | p.Trp1284Ser | NaV1.1 m | c.4970G>A | p.Arg1657His | NaV1.6 | c.4398C>A | p.Asn1466Lys |
| NaV1.1 s | c.3860T>C | p.Leu1287Pro | NaV1.1 m | c.5018T>G | p.Ile1673Thr | NaV1.6 | c.4400T>G | p.Phe1467Cys |
| NaV1.1 s | c.3862G>A | p.Asp1288Asn | NaV1.1 m | c.5054C>T | p.Ala1685Val | NaV1.6 | c.4423G>A | p.Gly1475Arg |
| NaV1.1 s | c.3899C>G | p.Thr1300Arg | NaV1.1 m | c.5216C>T | p.Pro1739Leu | NaV1.6 | c.4427G>A | p.Gly1476Asp |
| NaV1.1 s | c.3924A>T | p.Glu1308Asp | NaV1.1 m | c.5225C>G | p.Asp1742Gly | NaV1.6 | c.4435A>G | p.Ile1479Val |
| NaV1.1 s | c.3938A>T | p.Lys1313Ile | NaV1.1 m | c.5295T>A | p.Phe1765Leu | NaV1.6 | c.4447G>A | p.Glu1483Lys |
| NaV1.1 s | c.3946A>T | p.Arg1316Trp | NaV1.1 m | c.5305T>C | p.Tyr1769His | NaV1.6 | c.4474A>G | p.Met1492Val |
| NaV1.1 s | c.3946A>G | p.Arg1316Gly | NaV1.1 m | c.5309T>A | p.Ile1770Asn | NaV1.6 | c.4493A>T | p.Lys1498Met |
| NaV1.1 s | c.3948G>T | p.Arg1316Ser | NaV1.1 m | c.5383G>A | p.Glu1795Lys | NaV1.6 | c.4501C>A | p.Gln1501Lys |
| NaV1.1 s | c.3953T>G | p.Leu1318Arg | NaV1.1 m | c.5561T>C | p.Leu1854Ser | NaV1.6 | c.4594A>T | p.Ile1532Phe |
| NaV1.1 s | c.3959C>T | p.Ala1320Val | NaV1.1 m | c.5567T>C | p.Met1856Thr | NaV1.6 | c.4608G>A | p.Met1536Ile |
| NaV1.1 s | c.3965G>T | p.Arg1322Ile | NaV1.1 m | c.5569G>T | p.Val1857Leu | NaV1.6 | c.4748T>C | p.Ile1583Thr |
| NaV1.1 s | c.3974G>C | p.Arg1325Thr | NaV1.1 m | c.5596G>T | p.Asp1866Tyr | NaV1.6 | c.4750G>A | p.Gly1584Arg |
| NaV1.1 s | c.3976G>C | p.Ala1326Pro | NaV1.1 m | c.5600T>C | p.Ile1867Thr | NaV1.6 | c.4774G>C | p.Val1592Leu |
| NaV1.1 s | c.3977C>A | p.Ala1326Asp | NaV1.1 m | c.5600T>C | p.Ile1867Thr | NaV1.6 | c.4780A>C | p.Ile1594Leu |
| NaV1.1 s | c.3982T>C | p.Ser1328Pro | NaV1.1 m | c.5681C>T | p.Pro1894Leu | NaV1.6 | c.4787C>G | p.Ser1596Cys |
| NaV1.1 s | c.3995G>A | p.Gly1332Glu | NaV1.1 m | c.5752T>A | p.Ser1918Thr | NaV1.6 | c.4793T>C | p.Val1598Ala |
| NaV1.1 s | c.4003G>A | p.Val1335Met | NaV1.1 m | c.5768A>G | p.Gln1923Arg | NaV1.6 | c.4814T>G | p.Ile1605Arg |
| NaV1.1 s | c.4016C>T | p.Ala1339Val | NaV1.1 m | c.5779A>G | p.Arg1927Gly | NaV1.6 | c.4840A>G | p.Thr1614Ala |
| NaV1.1 s | c.4032T>G | p.Ile1344Met | NaV1.1 m | c.5864T>C | p.Ile1955Thr | NaV1.6 | c.4850G>A | p.Arg1617Gln |
| NaV1.1 s | c.4049T>G | p.Val1350Gly | NaV1.1 m | c.5962C>T | p.Arg1988Trp | NaV1.6 | c.4850G>C | p.Arg1617Pro |
| NaV1.1 s | c.4061G>A | p.Cys1354Tyr | NaV1.2 | c.34G>A | p.Asp12Asn | NaV1.6 | c.4862T>G | p.Leu1621Trp |
| NaV1.1 s | c.4064T>C | p.Leu1355Pro | NaV1.2 | c.82C>T | p.Arg28Cys | NaV1.6 | c.4873G>A | p.Gly1625Arg |
| NaV1.1 s | c.4072T>C | p.Trp1358Arg | NaV1.2 | c.106A>G | p.Arg36Gly | NaV1.6 | c.4889T>C | p.Leu1630Pro |
| NaV1.1 s | c.4073G>C | p.Trp1358Ser | NaV1.2 | c.209C>T | p.Pro70Leu | NaV1.6 | c.4925T>G | p.Phe1642Cys |
| NaV1.1 s | c.4086C>G | p.Ser1362Arg | NaV1.2 | c.245A>G | p.Asp82Gly | NaV1.6 | c.4948G>A | p.Ala1650Thr |
| NaV1.1 s | c.4088T>A | p.Ile1363Asn | NaV1.2 | c.396T>A | p.Asn132Lys | NaV1.6 | c.4949C>T | p.Ala1650Val |
| NaV1.1 s | c.4091T>A | p.Met1364Lys | NaV1.2 | c.408G>T | p.Met136Ile | NaV1.6 | c.5261T>C | p.Phe1754Ser |
| NaV1.1 s | c.4093G>A | p.Gly1365Ser | NaV1.2 | c.506A>G | p.Glu169Gly | NaV1.6 | c.5302A>G | p.Asn1768Asp |
| NaV1.1 s | c.4094G>A | p.Gly1365Asp | NaV1.2 | c.514A>G | p.Ile172Val | NaV1.6 | c.5401C>G | p.Gln1801Glu |
| NaV1.1 s | c.4096G>A | p.Val1366Ile | NaV1.2 | c.562C>T | p.Arg188Trp | NaV1.6 | c.5421G>C | p.Lys1807Asn |
| NaV1.1 s | c.4101T>A | p.Asn1367Lys | NaV1.2 | c.605C>T | p.Ala202Val | NaV1.6 | c.5483A>T | p.Met1828Val |
| NaV1.1 s | c.4108G>C | p.Ala1370Pro | NaV1.2 | c.620T>C | p.Phe207Ser | NaV1.6 | c.5555C>T | p.Thr1852Ile |
| NaV1.1 s | c.4109C>T | p.Ala1370Val | NaV1.2 | c.623T>A | p.Val208Glu | NaV1.6 | c.5558A>G | p.Lys1853Arg |
| NaV1.1 s | c.4112G>T | p.Gly1371Val | NaV1.2 | c.632G>A | p.Gly211Asp | NaV1.6 | c.5597G>A | p.Arg1866Gln |
| NaV1.1 s | c.4126T>C | p.Cys1376Arg | NaV1.2 | c.634A>G | p.Asn212Asp | NaV1.6 | c.5610A>C | p.Glu1870Asp |
| NaV1.1 s | c.4132A>C | p.Asn1378His | NaV1.2 | c.638T>A | p.Val213Asp | NaV1.6 | c.5614C>T | p.Arg1872Trp |
| NaV1.1 s | c.4133A>C | p.Asn1378Thr | NaV1.2 | c.653C>A | p.Thr218Lys | NaV1.6 | c.5615G>A | p.Arg1872Gln |
| NaV1.1 s | c.4153T>G | p.Phe1385Val | NaV1.2 | c.658A>G | p.Arg220Gly | NaV1.6 | c.5615G>T | p.Arg1872Leu |
| NaV1.1 s | c.4168G>A | p.Val1390Met | NaV1.2 | c.668G>A | p.Arg223Gln | NaV1.6 | c.5615G>C | p.Arg1872Pro |
| NaV1.1 s | c.4172A>G | p.Asn1391Ser | NaV1.2 | c.680C>T | p.Thr227Ile | NaV1.6 | c.5630A>G | p.Asn1877Ser |
| NaV1.1 s | c.4178A>C | p.His1393Pro | NaV1.2 | c.707C>G | p.Thr236Ser | NaV1.6 | c.5719C​>T | p.Arg1907Trp |
| NaV1.1 s | c.4181C>T | p.Thr1394Ile | NaV1.2 | c.710T>A | p.Ile237Asn |  |  |  |
| NaV1.1 s | c.4183G>T | p.Asp1396Tyr | NaV1.2 | c.718G>T | p.Ala240Ser |  |  |  |
| NaV1.1 s | c.4186T>G | p.Cys1396Gly | NaV1.2 | c.752T>C | p.Val251Ala |  |  |  |
| NaV1.1 s | c.4216G>A | p.Ala1406Thr | NaV1.2 | c.754A>G | p.Met252Val |  |  |  |
| NaV1.1 s | c.4228A>T | p.Asn1410Tyr | NaV1.2 | c.773G>C | p.Cys258Ser |  |  |  |
| NaV1.1 s | c.4240A>T | p.Asn1414Tyr | NaV1.2 | c.781G>A | p.Val261Met |  |  |  |
