# Supplementary material for: Variable patterns of mutation density among NaV1.1, NaV1.2 and NaV1.6 point to channel-specific functional differences associated with childhood epilepsy
Source: PLoS One. 2020 Aug 26;15(8):e0238121. doi: 10.1371/journal.pone.0238121 (PMC7449494; doi:10.1371/journal.pone.0238121)
Supplement: S3 Table — (DOCX) [file pone.0238121.s007.docx]

**S3 Table.** Breakdown of regions and their functionality based on previous literature and the ultimate grouping they were placed in.

| **Region** | **Role in Channel Activity** | **Functionality** |
| --- | --- | --- |
| N-terminus | Some regions implicated in regulating inactivation, biphasic nature reported in Na_V_1.1 SMEI | N/A |
| S1-S3 | Creates vestibule for S4 to slide into | Low |
| S3-S4 | Contains binding sites for channel modulation | N/A |
| S4 | Voltage-sensor | High |
| S4-S5 | Communicates movement of S4 to pore domain | High |
| S5 | Part of pore-forming domain | High |
| S5-S6 | Contains selectivity filter | High |
| S6 | Lines the pore and contains activation gate | High |
| DI-DII | Some sites associated with phosphorylation | Low |
| DII-DIII | No reported function | Low |
| DIII-DIV | Contains inactivation gate | High |
| C-terminus | Some regions implicated in regulating inactivation, biphasic nature reported in Na_V_1.1 | N/A |
