# Supplementary material for: Variable patterns of mutation density among NaV1.1, NaV1.2 and NaV1.6 point to channel-specific functional differences associated with childhood epilepsy
Source: PLoS One. 2020 Aug 26;15(8):e0238121. doi: 10.1371/journal.pone.0238121 (PMC7449494; doi:10.1371/journal.pone.0238121)
Supplement: S4 Table — (DOCX) [file pone.0238121.s008.docx]

**S4 Table**. Statistically significant (p<0.05) OR values for patient variant burden by domain for Na_V_1.1, Na_V_1.2, and Na_V_1.6.

| **Domain** | **S1** | **S1-S2** | **S2** | **S2-S3** | **S3** | **S3-S4** | **S4** | **S4-S5** | **S5** | **S5-S6** | **S6** |
| --- | --- | --- | --- | --- | --- | --- | --- | --- | --- | --- | --- |
| DI |  |  |  |  | 1.1 |  |  | 1.6 | 1.2, 1.6 | 1.1 | 1.1, 1.2, 1.6 |
| DII |  |  |  |  |  |  | 1.1, 1.2, 1.6 | 1.2, 1.6 | 1.2, 1.6 | 1.1 | 1.1, 1.2 |
| DIII | 1.1 |  |  |  |  |  | 1.1, 1.2 | 1.2, 1.6 | 1.1 | 1.1 | 1.1 |
| DIV |  |  |  |  | 1.2, 1.6 |  | 1.1, 1.2, 1.6 | 1.1, 1.2, 1.6 | 1.1 | 1.1 | 1.1, 1.2 |

Filled cells indicate Fisher test p-value <0.0001. Single statistically significant OR value for public variant burden in Na_V_1.1 S1-S2_DIV_ is not shown
