# Supplementary material for: Variable patterns of mutation density among NaV1.1, NaV1.2 and NaV1.6 point to channel-specific functional differences associated with childhood epilepsy
Source: PLoS One. 2020 Aug 26;15(8):e0238121. doi: 10.1371/journal.pone.0238121 (PMC7449494; doi:10.1371/journal.pone.0238121)
Supplement: S5 Table — (DOCX) [file pone.0238121.s009.docx]

**S5 Table**. Na_V_1.1 mutations by segment and domain for the A. patient database and for the B. public database.

| **Na_V_1.1 MS - Unique Variants (Dravet)** | | | | | |  |  |  |  |  |  |  |
| --- | --- | --- | --- | --- | --- | --- | --- | --- | --- | --- | --- | --- |
|  | nondomain | S1 | S2 | S3 | S4 | S5 | S6 | S1-2 | S2-3 | S3-4 | S4-5 | S5-6 |
| C | 26 |  |  |  |  |  |  |  |  |  |  |  |
| DI |  | 4 | 6 | 10 | 10 | 4 | 9 | 0 | 4 | 2 | 5 | 51 |
| DII |  | 3 | 2 | 5 | 8 | 7 | 12 | 2 | 0 | 1 | 2 | 34 |
| DIII |  | 8 | 7 | 4 | 11 | 16 | 9 | 6 | 0 | 1 | 4 | 44 |
| DIV |  | 7 | 7 | 4 | 11 | 11 | 17 | 0 | 3 | 3 | 13 | 24 |
| N | 26 |  |  |  |  |  |  |  |  |  |  |  |
| DI-II | 11 |  |  |  |  |  |  |  |  |  |  |  |
| DII-DIII | 11 |  |  |  |  |  |  |  |  |  |  |  |
| DIII-DIV | 11 |  |  |  |  |  |  |  |  |  |  |  |
|  |  |  |  |  |  |  |  |  |  |  |  |  |
| **Na_V_1.1 MS - Unique Variants (Mild)** | | | | | |  |  |  |  |  |  |  |
|  | nondomain | S1 | S2 | S3 | S4 | S5 | S6 | S1-2 | S2-3 | S3-4 | S4-5 | S5-6 |
| C | 12 |  |  |  |  |  |  |  |  |  |  |  |
| DI |  | 0 | 0 | 1 | 3 | 0 | 3 | 1 | 1 | 0 | 0 | 4 |
| DII |  | 0 | 0 | 0 | 1 | 0 | 4 | 3 | 0 | 0 | 0 | 2 |
| DIII |  | 1 | 2 | 0 | 3 | 2 | 0 | 2 | 0 | 2 | 1 | 7 |
| DIV |  | 1 | 1 | 2 | 3 | 2 | 3 | 0 | 2 | 0 | 0 | 2 |
| N | 8 |  |  |  |  |  |  |  |  |  |  |  |
| DI-II | 4 |  |  |  |  |  |  |  |  |  |  |  |
| DII-DIII | 1 |  |  |  |  |  |  |  |  |  |  |  |
| DIII-DIV | 1 |  |  |  |  |  |  |  |  |  |  |  |
|  |  |  |  |  |  |  |  |  |  |  |  |  |
| **Na_V_1.1 GNOMAD (this table with only single count for each mutational position)** | | | | | | | | | | | |  |
|  | nondomain | S1 | S2 | S3 | S4 | S5 | S6 | S1-2 | S2-3 | S3-4 | S4-5 | S5-6 |
| C | 104 |  |  |  |  |  |  |  |  |  |  |  |
| DI |  | 2 | 5 | 1 | 4 | 3 | 3 | 0 | 3 | 2 | 3 | 36 |
| DII |  | 7 | 2 | 4 | 2 | 5 | 4 | 6 | 5 | 3 | 5 | 4 |
| DIII |  | 1 | 6 | 7 | 3 | 4 | 3 | 6 | 0 | 1 | 4 | 10 |
| DIV |  | 10 | 7 | 7 | 2 | 5 | 2 | 8 | 2 | 2 | 0 | 14 |
| N | 46 |  |  |  |  |  |  |  |  |  |  |  |
| DI-II | 148 |  |  |  |  |  |  |  |  |  |  |  |
| DII-DIII | 100 |  |  |  |  |  |  |  |  |  |  |  |
| DIII-DIV | 8 |  |  |  |  |  |  |  |  |  |  |  |
