# Supplementary material for: Variable patterns of mutation density among NaV1.1, NaV1.2 and NaV1.6 point to channel-specific functional differences associated with childhood epilepsy
Source: PLoS One. 2020 Aug 26;15(8):e0238121. doi: 10.1371/journal.pone.0238121 (PMC7449494; doi:10.1371/journal.pone.0238121)
Supplement: S6 Table — (DOCX) [file pone.0238121.s010.docx]

**S6 Table**. Na_V_1.2 mutations by segment and domain for the A. patient database and for the B. public database.

| **Na_V_1.2 Patient Missense - Unique Variants** | | | | |  |  |  |  |  |  |  |  |
| --- | --- | --- | --- | --- | --- | --- | --- | --- | --- | --- | --- | --- |
|  | nondomain | S1 | S2 | S3 | S4 | S5 | S6 | S1-2 | S2-3 | S3-4 | S4-5 | S5-6 |
| C | 18 |  |  |  |  |  |  |  |  |  |  |  |
| DI |  | 2 | 2 | 3 | 4 | 6 | 4 |  | 1 | 3 | 3 | 5 |
| DII |  | 3 | 1 | 2 | 6 | 8 | 5 | 1 |  |  | 7 | 6 |
| DIII |  | 2 | 2 | 2 | 6 | 2 | 3 |  |  |  | 9 | 4 |
| DIV |  | 4 | 4 | 7 | 12 | 2 | 4 |  | 1 | 2 | 5 | 3 |
| N | 5 |  |  |  |  |  |  |  |  |  |  |  |
| DI-DII | 12 |  |  |  |  |  |  |  |  |  |  |  |
| DII-DIII | 12 |  |  |  |  |  |  |  |  |  |  |  |
| DIII-DIV | 5 |  |  |  |  |  |  |  |  |  |  |  |
|  |  |  |  |  |  |  |  |  |  |  |  |  |
| **Na_V_1.2 GNOMAD (this table with only single count for each mutational position)** | | | | | | | | | | | |  |
|  | nondomain | S1 | S2 | S3 | S4 | S5 | S6 | S1-2 | S2-3 | S3-4 | S4-5 | S5-6 |
| C | 76 |  |  |  |  |  |  |  |  |  |  |  |
| DI |  | 5 | 4 | 5 | 2 |  | 1 | 1 | 6 | 1 | 2 | 27 |
| DII |  | 3 | 2 | 4 | 1 | 1 | 2 | 3 |  |  |  | 8 |
| DIII |  | 6 | 7 | 4 | 2 | 2 | 3 | 6 | 1 | 2 | 3 | 17 |
| DIV |  | 3 | 4 | 3 | 1 | 1 | 1 | 2 |  |  |  | 5 |
| N | 44 |  |  |  |  |  |  |  |  |  |  |  |
| DI-DII | 115 |  |  |  |  |  |  |  |  |  |  |  |
| DII-DIII | 96 |  |  |  |  |  |  |  |  |  |  |  |
| DIII-DIV | 7 |  |  |  |  |  |  |  |  |  |  |  |
