# Supplementary material for: Variable patterns of mutation density among NaV1.1, NaV1.2 and NaV1.6 point to channel-specific functional differences associated with childhood epilepsy
Source: PLoS One. 2020 Aug 26;15(8):e0238121. doi: 10.1371/journal.pone.0238121 (PMC7449494; doi:10.1371/journal.pone.0238121)
Supplement: Attachment — Submitted filename: Review of PONE-D-20-07729.pdf [file pone.0238121.s012.pdf]

**Review of PONE-D-20-07729 entitled "Variable patterns of mutation density among NaV1.1, NaV1.2 and NaV1.6 point to channel-specific functional differences associated with childhood epilepsy"**

## Summary of the research and overall impression

This paper describes and compares the distribution of mutations in NaV1.1, 1.2 and 1.6 channels found in patients diagnosed with epilepsy and in the general public research data bases. Based on the location of the mutations within the sodium channels and the presence of the mutations in patient and public databases, conclusions are drawn as to whether the mutations are pathogenic (detected in patient databases), are benign (present only in the public data base) or are possibly embryonic lethal/non-functional (no mutations detected in both patient and public databases).

There are no overall issues with the data or conclusions. The authors clearly describe the data bases used in the statistical analysis and point out the potential shortcomings. It is an interesting approach to identifying differences between the various sodium channels with respect to the relationships between the function/role of the sodium channel in neuronal firing and the region/effects of a given mutation. The results suggest that the same mutation in highly conserved regions of the sodium channels may not have the same effect on neuronal activity or even result in epilepsy for a given sodium channel. This approach would allow researchers to study more clinically-relevant mutations in ion channels and give greater insight into the relationship between ion channel structure/function and consequences on neuronal activity.

One issue, that was beyond the scope of the present study, is the possibility that mutations/SNPs in other proteins that are important for sodium channel trafficking/function (e.g. presence of beta subunit polymorphisms) may interact with the sodium channel mutations resulting in either changes in function and an epileptic phenotype or compensate for the effect of the sodium channel mutation. Is it possible, for example, that a mutation present only in the public database is a result of a mutant beta subunit that counteracts the deleterious effect of the sodium channel mutation and, as a result, does not result in an epileptic phenotype?

Given the increasing push towards personalized medicine in drug treatment, where a patient's stem cells are converted to neurons and then characterized, this type of statistical analysis may become more common place. Especially in the area of drug development. In a recent commentary on the clinical failure of a topical analgesic targeting NaV1.7 (Hesselink JMK (2018) Topical NaV1.7 channel blocker failed in postherpetic Neuralgia (PHN): lessons to learn. Gen Med Open, DOI: 10.15761/GMO.1000126) a subset of patients with a specific NaV1.7 gain of function mutation did show some analgesic affect greater than that of a placebo. It would be interesting to expand the authors' approach to determine if there is an interaction between the effect (or lack of an effect) of a drug and distribution of mutations (although the amount of existing data may not allow for statistical analysis). This could give insight into how to better develop new drugs or utilize existing drugs for treatment of patients with specific mutations.
