# Supplementary material for: Variable patterns of mutation density among NaV1.1, NaV1.2 and NaV1.6 point to channel-specific functional differences associated with childhood epilepsy
Source: PLoS One. 2020 Aug 26;15(8):e0238121. doi: 10.1371/journal.pone.0238121 (PMC7449494; doi:10.1371/journal.pone.0238121)
Supplement: Attachment — Submitted filename: Response to Reviewers.docx [file pone.0238121.s013.docx]

**Reviewer #1:**

*1a) The authors assessed the mutational burden in different regions of the NaV channels considering (1) the excess variants in the public databases and (2) the cumulative distribution of variants throughout the different genes.* ***However, this approach is quite arbitrary****.*

**Response: We not sure what the reviewer means by “arbitrary”; however, the cumulative function distribution contains all the information necessary for the statistical tests (i.e., sufficient for all the statistical tests performed in these analyses).**

*1b) Moreover, there was a* ***clear discrepancy*** *in the choice of the different patients’ database used for this study (i.e., Japanese patients with Dravet syndrome (DS); the literature data for the NaV1.2 missense variants, the patient’s registry for SCN8A mutations. This choice must be discussed as it did potentially affect the results, at least in part. For instance, Nav 1.1 mutations are associated with a spectrum of severity, ranging from autosomal dominant GEFS+ to de novo mutations causing DS. Thus, as honestly discussed by the authors, the adoption of the Japanese register did exclude a priori all the milder form of epilepsy associated with mutations in SCN1A.*

**Response: We completely agree and thank the reviewer for noting the discrepancy. We have now performed an analysis of ALL SCN1A variants—including an additional 94 patients with milder symptoms in addition to the the Dravet Syndrome patients. While including the milder patients did not alter our main results in a significant way (i.e., variants in the milder group tended to be found in similar segments, domains, linkers and loops as variants with Dravet Syndrome), we did discover that variants associated with these patients tended to accumulate more in the N- and C- terminus. This raises new questions abou the functionality of these regions of the gene, which we now discuss in the last section of the manuscript.**

**Future analyses that include greater numbers of variants will have additional statistical power to identify sites in the channel with patient burden. These studies will need to include associations with patient phenotype (i.e., along the spectrum of disease outcome).**

*2) Overall, pathogenic variants in these genes have been associated with a wide spectrum of epilepsy phenotypes, ranging from benign epilepsies to epileptic encephalopathies with variable severity. Furthermore, a few patients with intellectual disability (ID) or movement disorders without epilepsy have been reported (see, for instance, Johannesen KM, The spectrum of intermediate SCN8A-related epilepsy. Epilepsia. 2019;60(5):830-844).*

**Response: We now refer to this broader spectrum and cite the Johannessen et al. reference. And restate the last sentence of the first paragraph of the introduction to clarify this point.**

*3) The case of NaV 1.1 variants is even more complicated if we consider that some variants can promote inclusion of a "poison" exon that leads to reduced amounts of full-length SCN1A protein, a mechanism is likely to be broadly relevant to human disease (Carvill GL, Aberrant Inclusion of a Poison Exon Causes Dravet Syndrome and Related SCN1A-Associated Genetic Epilepsies. Am J Hum Genet. 2018;103(6):1022-1029).*

**Response: We agree that this is an interesting finding. However, we feel it is beyond the scope of this study our focus on variants across the entire gene in patient and public databases while variants in the abovementioned exon occur outside the annotated coding regions..**

**Reviewer #2:**

*There are no overall issues with the data or conclusions. The authors clearly describe the data bases used in the statistical analysis and point out the potential shortcomings. It is an interesting approach to identifying differences between the various sodium channels with respect to the relationships between the function/role of the sodium channel in neuronal firing and the region/effects of a given mutation. This approach would allow researchers to study more clinically-relevant mutations in ion channels and give greater insight into the relationship between ion channel structure/function and consequences on neuronal activity.*
